# Advancing Prostate Cancer Assessment: A Biparametric MRI (T2WI and DWI/ADC)-Based Radiomic Approach to Predict Tumor–Stroma Ratio

**DOI:** 10.3390/diagnostics15212722

**Published:** 2025-10-27

**Authors:** Jiangqin Ma, Xiling Gu, Zhonglin Zhang, Jun Chen, Yunfan Liu, Yang Qiu, Guangyong Ai, Xuxiang Jia, Zhenghao Li, Bo Xiang, Xiaojing He

**Affiliations:** 1 Department of Radiology, The Second Affiliated Hospital of Chongqing Medical University, Chongqing 400010, China; 2 Department of Pathology, The Second Affiliated Hospital of Chongqing Medical University, Chongqing 400010, China; 3 Beijing Institute of Technology, Beijing 100081, China; 4 Chongqing Institute of Green and Intelligent Technology, Chinese Academy of Sciences, Chongqing 400714, China; 5 Department of Radiology, Yongchuan Hospital of Chongqing Medical University, Chongqing 402115, China

**Keywords:** prostate cancer, tumor–stroma ratio, radiomics, biparametric MRI, machine learning

## Abstract

**Objectives:** This study aimed to develop and validate a biparametric MRI (bpMRI)-based radiomics model for the noninvasive prediction of tumor–stroma ratio (TSR) in prostate cancer (PCa). Additionally, we sought to explore lesion distribution patterns in the peripheral zone (PZ) and transition zone (TZ) to provide deeper insights into the biological behavior of PCa. **Methods**: This multicenter retrospective study included 223 pathologically confirmed PCa patients, with 146 for training and 39 for internal validation at Center 1, and 38 for external testing at Center 2. All patients underwent preoperative bpMRI (T2WI, DWI acquired with a b-value of 1400 s/mm^2^, and ADC maps), with TSR histopathologically quantified. Regions of interest (ROIs) were manually segmented on bpMRI images using ITK-SNAP software (version 4.0.1), followed by high-throughput radiomic feature extraction. Redundant features were eliminated via Spearman correlation analysis and least absolute shrinkage and selection operator (LASSO) regression. Five machine learning (ML) classifiers—Logistic Regression (LR), Support Vector Machine (SVM), BernoulliNBBayes, Ridge, and Stochastic Gradient Descent (SGD)—were trained and optimized. Model performance was rigorously evaluated using receiver operating characteristic (ROC) curves and decision curve analysis (DCA). **Results:** The Ridge demonstrated superior diagnostic performance, achieving AUCs of 0.846, 0.789, and 0.745 in the training, validation, and test cohorts, respectively. Lesion distribution analysis revealed no significant differences between High-TSR and Low-TSR groups (*p* = 0.867), suggesting that TSR may not be strongly associated with zonal localization. **Conclusions:** This exploratory study suggests that a bpMRI-based radiomic model holds promise for noninvasive TSR estimation in prostate cancer and may provide complementary insights into tumor aggressiveness beyond conventional pathology.

## 1. Introduction

The tumor microenvironment (TME) is a sophisticated and dynamic ecosystem playing a pivotal role in cancer progression. The TME consists of a variety of cellular components, such as cancer-associated fibroblasts (CAFs), immune cells, endothelial cells, pericytes, adipocytes, and mesenchymal stem cells (MSCs), as well as non-cellular components including the extracellular matrix (ECM), cytokines, exosomes, soluble proteins, and metabolites [1]. Within the TME, the tumor stroma stands out as the core component which consists of activated CAFs, immune cells, endothelial cells, and acellular components like the ECM and cancer-specific vascular systems [2]. This key component not only supports but also actively enhances the cancer’s lifecycle, influencing its initiation, growth, metastasis, and response to therapy.

The tumor–stroma ratio (TSR) has gained recognition as a critical concept for quantifying the tumor stroma. TSR is defined as the ratio of tumor cells to the surrounding stroma within a tumor. Its significant role as a prognostic indicator is increasingly acknowledged in various cancers, including colon [3], nasopharyngeal [4], and breast cancer [5]. In prostate cancer (PCa), the stroma has been identified as significantly correlated with invasiveness [6] and its role as an independent prognostic factor for predicting biochemical recurrence has been highlighted [7,8]. Additionally, tumor stroma is strongly associated with key pathological parameters, such as tumor size [9] and depth of invasion [10], positions it as a promising marker for routine histologic prognosis in PCa.

The conventional approach to TSR assessment, which relies on invasive histopathological methods, presents significant challenges, including patient discomfort and procedural risks [11,12]. Recognizing these limitations, researchers have made strides in non-invasive TSR evaluation. Studies correlating apparent diffusion coefficient (ADC) values [13,14] and stromal content have been promising, and intravoxel incoherent motion (IVIM) imaging [15,16] has shown potential in reflecting stromal characteristics. Despite these advancements, the reliance on specific magnetic resonance imaging (MRI) quantitative parameters introduces challenges related to signal variability and image noise [17,18]. Moreover, these parameters typically represent only a portion of the complex tissue architecture. Recently, radiomics has emerged as an innovative non-invasive imaging analysis technique, offering a comprehensive view of tissue characteristics by extracting extensive imaging features [19]. Preliminary studies in radiomics applied to TSR assessment have shown promising results. For instance, the prediction of TSR in rectal cancer using a rad-score achieved notable predictive outcomes with an area under the receiver operating characteristic (ROC) curve (AUC) of 0.787 [14]. However, radiomics methods rely on manual feature selection and linear modeling, making it difficult to fully capture the intratumoral heterogeneity. Machine learning (ML) methods, as data-driven tools, can efficiently identify complex nonlinear relationships within high-dimensional radiomic features [20,21]. By automating feature selection and classification modeling, ML significantly improves both the predictive performance and generalizability of models. For example, in studies of pancreatic ductal adenocarcinoma using T1-weighted enhanced images, the extreme gradient boosting (XGBoost) classifier achieved robust predictive performance, with an AUC of 0.78 [22]. These findings collectively underscore the potential of radiomics–ML frameworks to enhance TSR estimation across multiple tumor types. However, their application in noninvasive TSR prediction for PCa remains underexplored and warrants further investigation.

Therefore, this study presents a novel bpMRI-based radiomic model, marking the first exploratory attempt to non-invasively predict the TSR in PCa, with its performance further validated in an external cohort. Unlike previous investigations that predominantly relied on a single imaging sequence or limited quantitative parameters, our study integrated information from T2WI, DWI, ADC maps, combined with advanced ML techniques to extract and analyze high-dimensional radiomic features. This comprehensive approach enabled a more nuanced characterization of TME heterogeneity. By overcoming the inherent limitations of invasive histopathological methods, the proposed framework offers a novel imaging-based tool to support individualized risk stratification and facilitate more precise clinical decision-making for patients with PCa. In addition, we analyzed the lesion distribution of the H-TSR and L-TSR groups in the peripheral zone (PZ) and transition zone (TZ), to further understand the biological behavior of PCa.

## 2. Materials and Methods

### 2.1. Patient Cohort and Recruitment

This study enrolled a total of 327 patients, comprising 267 individuals who underwent prostate MRI scans at the Center 1 from January 2020 to June 2023, and an additional 60 patients recruited from Center 2 between January 2021 and June 2023.

Patients were selected based on the following criteria: (a) Completion of preoperative MRI protocols, including T2-weighted imaging (T2WI), diffusion-weighted imaging (DWI), and ADC mapping; (b) Histopathological confirmation of PCa within three months following MRI examination.

**Figure 1:**
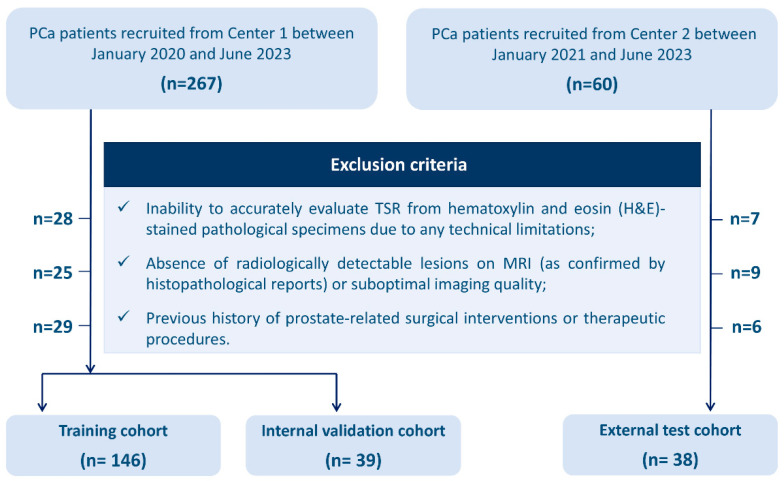
Flow chart of the patient selection process. H&E, Hematoxylin and eosin; PCa, prostate cancer; TSR, the tumor–stroma ratio.

Patients were excluded from the study based on the following parameters: (a) Inability to accurately evaluate TSR from hematoxylin and eosin (H&E)-stained pathological specimens due to any technical limitations; (b) Absence of radiologically detectable lesions on MRI (as confirmed by histopathological reports) or suboptimal imaging quality; (c) Previous history of prostate-related surgical interventions or therapeutic procedures. The comprehensive screening algorithm for study population selection is illustrated in Figure 1.

Demographic characteristics and clinical parameters were systematically collected for all participants, including age, prostate-specific antigen (PSA) levels, free PSA (fPSA) concentrations, fPSA/PSA ratio, prostate volume, and PSA density (PSAD).

This retrospective study was approved by the Institutional Ethics Committee of the Second Affiliated Hospital of Chongqing Medical University (Approval No. 130/2024). The requirement for informed consent was waived by the ethics committees due to the retrospective nature of the study.

### 2.2. Pathological Evaluation

A senior pathologist (G.X.L) with two decades of specialization in prostate pathology reviewed and assessed the H&E-stained tissue sections of primary PCa. The TSR was quantified as the proportion of stromal cells within the tumor microenvironment, specifically focusing on regions demonstrating the highest Gleason score (GS) in the biopsy specimens [3].

**Figure 2:**
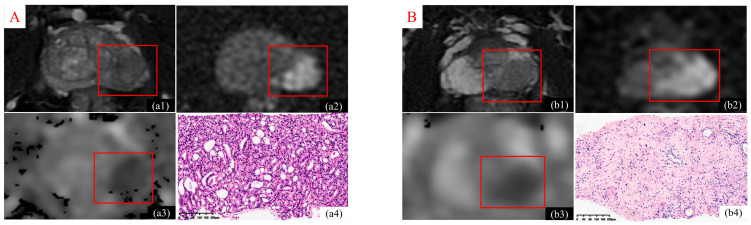
Representative biparametric MRI and histopathological features of PCa with high and low TSR. (**A**): (**a1**–**a3**) illustrate axial T2WI, DWI, and ADC maps, respectively, from a 63-year-old male diagnosed with PCa exhibiting a high TSR (≥50%). Panel (**a4**) displays the corresponding H&E–stained histological section, indicating low stromal content. (**B**): (**b1**–**b3**) demonstrate T2WI, DWI, and ADC maps of a 59-year-old male patient with low TSR (<50%). The matched histopathological section (**b4**) reveals a comparatively greater tumor epithelial component with high stromal content.

Based on TSR quantification, patients were stratified into two distinct cohorts: those with a stromal cell composition ≥50% were classified into the high-stroma group (indicative of low TSR, or L-TSR), while those with stromal cell composition <50% were categorized into the low-stroma group (indicative of high TSR, or H-TSR). A schematic representation of BpMR imaging and corresponding histopathological specimens from PCa patients with varying TSRs is presented in Figure 2.

### 2.3. MRI Protocols

MRI acquisitions were performed at both centers using 3.0 T MRI scanners. At Center 1, imaging was conducted using the MAGNETOM Prisma scanner (SIEMENS Healthineers, Erlangen, Germany) coupled with an 18-channel phased-array coil. At Center 2, the MAGNETOM Verio scanner (SIEMENS Healthineers, Erlangen, Germany) equipped with a 6-channel phased-array coil was utilized. Scanning protocols adhered strictly to the PI-RADS v2.1 guidelines included T2WI, DWI, and ADC maps. At Center 1, DWI was acquired with b-values of 0, 50, 1000, and 1400 s/mm^2^, whereas at Center 2, b-values of 0, 50, 1000, 1400, and 2000 s/mm^2^ were applied. A b-value of 1400 s/mm^2^ was consistently used at both centers for radiomic feature extraction, with full acquisition parameters detailed in Supplementary Table S1. ADC maps were reconstructed using FUNCTOOL on the Advanced Workstation 4.6 (GE Medical Systems, Milwaukee, WI, USA), based on the b = 1000 s/mm^2^ images. DWI was acquired using an EPI sequence combined with parallel imaging and a reduced field-of-view protocol to shorten acquisition time, improve signal-to-noise ratio, and minimize motion-related artifacts. All images were independently assessed by experienced radiologists, and cases with severe artifacts precluding reliable lesion identification or ROI delineation were excluded. Only images of acceptable quality were retained to ensure the reliability of radiomic feature extraction.

### 2.4. Radiomic Feature Extraction

MRI datasets were anonymized and transferred from the Centricity PACS Version 6.0 SP (GE Healthcare, Chicago, IL, USA) to a dedicated offline workstation for segmentation and subsequent radiomic analysis. Lesion delineation was performed using ITK-SNAP software (version 4.0.1; www.itk-snap.org (accessed on 25 January 2024)). The delineation encompassed the entire solid tumor volume, with adjacent edema, cystic, or necrotic regions explicitly excluded to avoid interference from non-tumorous tissue signals. Lesion boundaries were primarily defined on T2WI by identifying hypointense regions, supplemented by hyperintensity on high b-value DWI and corresponding hypointense on ADC maps as critical auxiliary criteria. The ROIs on DWI and ADC maps were automatically co-registered to T2WI using ITK-SNAP software to ensure that subsequent feature extraction was performed within a unified spatial reference framework. To ensure boundary accuracy and consistency, all ROIs were cross-validated across the three imaging sequences. In cases of multifocal PCa, the largest lesion was selected for analysis, with cystic or necrotic regions explicitly excluded.

Three-dimensional lesion segmentation was performed in ITK-SNAP by a radiologist (Q.X.F.) with over 8 years of prostate MRI experience and independently validated by a senior radiologist (H.X.J.) with more than 15 years of expertise. Both radiologists were blinded to clinical outcomes and histopathological results, and any discrepancies in segmentation were resolved through consensus-based discussions. The resulting volume of interest (VOI) images were subsequently processed using the InferScholar platform (version 6.0, InferVision, Beijing, China) for radiomic feature extraction and analysis.

During the preprocessing stage, the N4ITK algorithm embedded in ITK-SNAP was first applied to correct for intensity non-uniformities, thereby reducing low-frequency bias field artifacts arising from magnetic field inhomogeneities. Subsequently, all images underwent intensity normalization using the Z-score method, with signal values standardized to the 0–1 range to minimize inter-patient and inter-scan variability. To further guarantee spatial consistency, a B-spline interpolation algorithm was employed for image resampling, achieving a standardized isotropic resolution of 1.0 mm × 1.0 mm × 1.0 mm. Radiomic features encompassing first-order statistical features, shape-based features, and texture features. Texture features were further subdivided into 5 categories: gray-level co-occurrence matrix (GLCM), gray-level run length matrix (GLRLM), gray-level size zone matrix (GLSZM), neighboring gray tone difference matrix (NGTDM), and gray-level dependence matrix (GLDM). Additionally, wavelet transformation was further applied to the images to enhance edge and texture characteristics, thereby ensuring that both high- and low-frequency information could be adequately captured.

### 2.5. Radiomics Feature Selection and Model Building

Interobserver agreement of all extracted features was evaluated using the intraclass correlation coefficient (ICC), only features demonstrating high reproducibility (ICC > 0.80) were retained for subsequent analysis. To mitigate multicollinearity among radiomic features, an initial Spearman’s correlation analysis was conducted to identify feature pairs with an average absolute correlation coefficient exceeding 0.85. These highly correlated features were deemed redundant and subsequently excluded from further analysis. To further refine the feature set and retaining features with preserved semantic interpretability, the Least Absolute Shrinkage and Selection Operator (LASSO) regression was employed. The regularization parameters for LASSO were optimized through 10-fold cross-validation, ensuring enhanced model stability, reduced overfitting risk, and minimized selection bias within the radiomics framework.

With the remaining relevant radiomics features, five ML models were developed: Logistic Regression (LR), Support Vector Machine (SVM), BernoulliNBBayes, Ridge, and Stochastic Gradient Descent (SGD). The optimal hyperparameters for each model were determined using 5-fold cross-validation on the training dataset using the grid search algorithm. The model exhibiting the highest cross-validation performance was then selected for further analysis, ensuring the robustness and reliability of the results.

Feature selection and radiomics signature development were exclusively conducted within the training cohort. Model efficacy was initially evaluated using an internal validation cohort, independent of the training process, followed by external validation on a separate test cohort. The entire workflow, including feature selection and model development, was implemented using Python 3.7.12 (Python Software Foundation, Wilmington, DE, USA).

**Figure 3:**
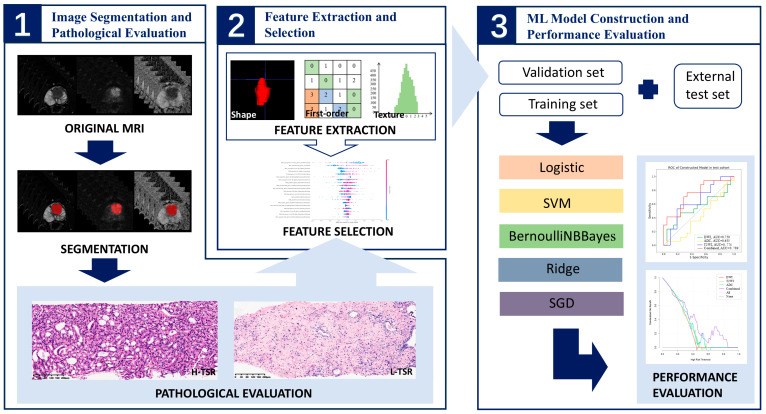
Work-flow chart of the study. From left to right, and top to bottom, the process unfolds as follows: First, patients were screened according to inclusion and exclusion criteria. Imaging data for all enrolled patients, including T2WI, DWI, and ADC maps, were retrieved from the Picture Archiving and Communication System (Centricity PACS Version 6.0 SP, GE Healthcare, Chicago, IL, USA). These images were then imported into ITK-Snap software, where lesions were manually delineated slice by slice. Next, the images were uploaded to the InferScholar (version 6.0, InferVision, Beijing, China) platform, and each patient was labeled based on their pathological results. Features were extracted using InferScholar, and the extracted features were imported into Python 3 software for screening of superior features through Spearman’s correlation analysis and the Least Absolute Shrinkage and Selection Operator (LASSO). Based on these selected, five predictive models were constructed: Logistic Regression (LR), Support Vector Machine (SVM), BernoulliNBBayes, Ridge, and Stochastic Gradient Descent (SGD). Finally, the performance of the models was evaluated by receiver operating characteristics (ROC), calibration curve, decision curves analysis (DCA), DeLong test and other indicators such as accuracy, sensitivity, and specificity.

A comprehensive schematic of the technical workflow is illustrated in Figure 3.

### 2.6. Statistical Analysis

Statistical analyses were performed using SPSS software (version 27.0; IBM Corporation, Armonk, NY, USA). A two-tailed *p*-value < 0.05 was considered statistically significant. The distribution of continuous variables was evaluated using the Shapiro–Wilk test. Data conforming to a normal distribution are reported as mean ± standard deviation (SD) and were analyzed using the independent samples *t*-test for comparisons between two groups or one-way analysis of variance (ANOVA) for comparisons among three or more groups. Non-normally distributed variables are presented as median and interquartile range (IQR; 25th–75th percentiles) and were compared using the Mann–Whitney U test for two groups or the Kruskal–Wallis test for multiple groups. Categorical variables were compared using chi-square test. To identify independent predictors of the TSR in PCa, binary logistic regression analysis was performed.

The predictive performance of the models was evaluated using receiver operating characteristic (ROC) curves, with areas under the curve (AUCs) and 95% confidence intervals (95% CIs) reported. Calibration curves were generated to assess the agreement between predicted probabilities and observed outcomes, supplemented by the Hosmer-Lemeshow test to evaluate statistical differences. Comparative analysis of ROC curves across models was conducted using the DeLong test. To further quantify improvements in diagnostic performance, integrated discrimination improvement (IDI) analysis was employed, offering a more refined metric for model comparison. Diagnostic metrics, including sensitivity, specificity, precision, accuracy, F1-score, and negative predictive value (NPV), were calculated alongside their 95% CIs to evaluate clinical utility. Decision curve analysis (DCA) was employed to quantify the net benefit of each model across a range of threshold probabilities. Shapley Additive Explanations (SHAP) analysis was performed to interpret feature contributions to the prediction of PCa TSR, enhancing model transparency and interpretability. Additionally, to further assess the presence of residual multicollinearity among the selected features, a correlation heatmap based on absolute Spearman correlation coefficients was constructed.

## 3. Results

### 3.1. Patient Characteristics

Patients from study center 1 were randomly stratified into a training cohort (*n* = 146) and an internal validation cohort (*n* = 39) in an approximate 8:2 ratio. Additionally, patients from center 2 were designated as the external test cohort (*n* = 38). The baseline demographic and clinical characteristics of the patients across the three cohorts are summarized in Table 1. The distribution of patients between the high tumor–stroma ratio (H-TSR) and low tumor–stroma ratio (L-TSR) groups was well-balanced, with 74 and 72 patients in the training cohort, 20 and 19 in the validation cohort, and 21 and 17 in the test cohort, respectively. Comparative analyses revealed no statistically significant differences in age or PI-RADS scores among the three cohorts. However, significant inter-cohort differences were noted in PSA, fPSA, fPSA/PSA ratio, prostate volume, and PSAD. To assess the independent association of these clinical variables with TSR status, a binary logistic regression analysis was performed. As presented in Supplementary Table S2, none of the evaluated clinical indicators emerged as independent predictors of TSR in patients with prostate cancer.

### 3.2. Feature Extraction and Selection

A total of 1746 radiomics features were extracted from T2WI, DWI, and ADC maps for each participant. Following rigorous feature selection, 28 highly impactful features were retained for model construction. Among these, 13 features were derived from ADC maps, 12 from DWI, and 3 from T2WI. The selected features comprised 5 first-order features and 23 texture features, including 7 GLDM features, 5 GLRLM features, 4 NGTDM features, 4 GLSZM features, and 3 GLCM features.

The SHAP summary plot ranks features by their importance, with the most critical feature at the top. The color gradient (red to blue) represents the magnitude of feature values, where red indicates high values and blue indicates low values. Positive SHAP values (right on the x-axis) correlate with favorable outcomes, while negative SHAP values (left on the x-axis) suggest unfavorable outcomes.

**Figure 4:**
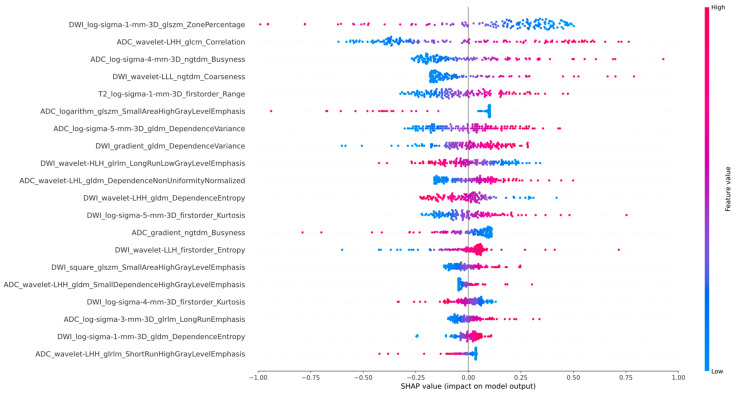
Shapley Additive exPlanations (SHAP) analysis for the Ridge in predicting PCa TSR.

**Figure 5:**
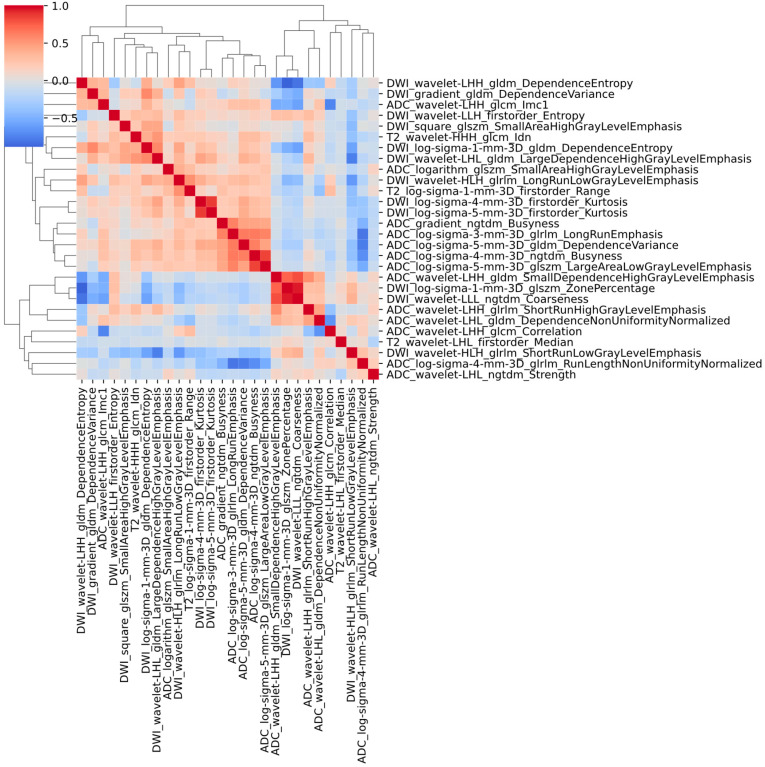
Heatmap of absolute Spearman correlation coefficients among the 28 selected radiomic features.

The SHAP plot for the Ridge is illustrated in Figure 4, with corresponding plots for the other models provided in Supplementary Figure S1. For the Ridge classifier, SHAP analysis revealed the three most influential radiomic features contributing to the model’s predictive performance: DWI_log-sigma-1-mm-3D_glszm_ZonePercentage, ADC_wavelet-LHH_glcm_Correlation, and ADC_log-sigma-4-mm-3D_ngtdm_Busyness. Notably, lower values of this feature (represented by blue dots) were strongly associated with a higher probability of favorable outcomes, indicating a significant negative correlation with positive prognosis. To further assess potential multicollinearity among input variables, a correlation heatmap based on absolute Spearman correlation coefficients was constructed (Figure 5). The heatmap demonstrated that most feature pairs exhibited low inter-feature correlations, indicating minimal redundancy across the selected radiomic descriptors. Only a small subset of feature pairs showed moderate correlations, thereby supporting the stability and interpretability of the model inputs.

### 3.3. Diagnostic Performance of the ML Models

In the single-sequence analyses, the predictive performance of the five machine learning models varied across imaging modalities. For the T2WI sequence, the AUCs ranged from 0.611 to 0.623 in the training cohort, 0.592 to 0.671 in the validation cohort, and 0.412 to 0.606 in the test cohort. For the DWI sequence, the corresponding AUCs were 0.656–0.771, 0.666–0.809, and 0.518–0.585, respectively. For the ADC sequence, the AUCs were relatively higher, ranging from 0.722 to 0.803 in the training cohort, 0.712 to 0.769 in the validation cohort, and 0.546 to 0.686 in the test cohort. Detailed information is provided in Supplementary Table S3.

**Figure 6:**
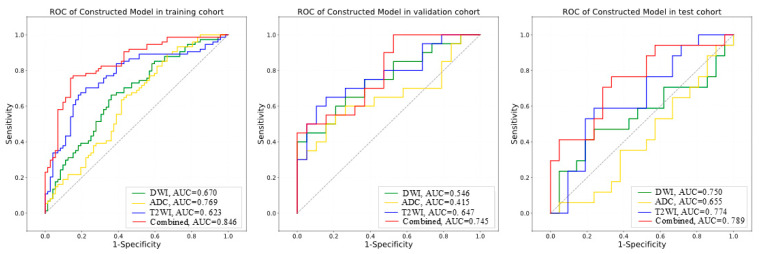
ROC curves for the Ridge model across the three cohorts.

In the combined-sequence analysis, the Ridge model exhibited the best predictive performance among the five ML models evaluated, achieving AUC values of 0.846 [95% CI: 0.782–0.909], 0.789 [95% CI: 0.648–0.931], and 0.745 [95% CI: 0.583–0.907] in the training, validation, and test cohorts, respectively. The corresponding accuracy values were 0.80 [95% CI: 0.796–0.807], 0.720 [95% CI: 0.695–0.741], and 0.68 [95% CI: 0.660–0.708]. A detailed performance comparison is presented in Table 2, with the corresponding ROC curves depicted in Figure 6. The AUC values of the other models are provided in Supplementary Table S4 and Supplementary Figure S2. The DeLong test revealed no statistically significant differences in AUC values among the five models across the validation and test cohorts (*p* > 0.05). Further details are provided in Supplementary Table S5. To further delineate relative model efficacy, IDI analysis was performed. The Ridge model consistently outperformed most comparator models across all cohorts in terms of discriminative capacity, with the exception of the SVM, for which no statistically significant advantage was observed in the external test cohort. Detailed IDI results are provided in Supplementary Table S6.

**Figure 7:**
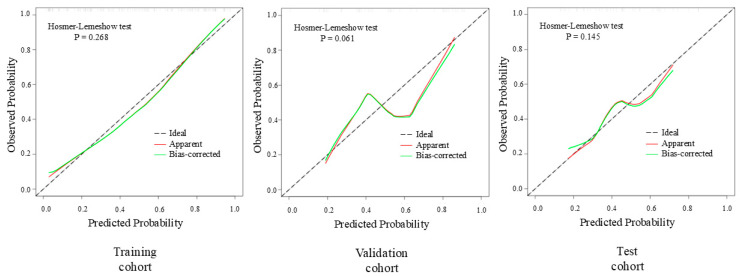
The calibration curves illustrate the calibration performance of the Ridge model across the three independent cohorts.

**Figure 8:**
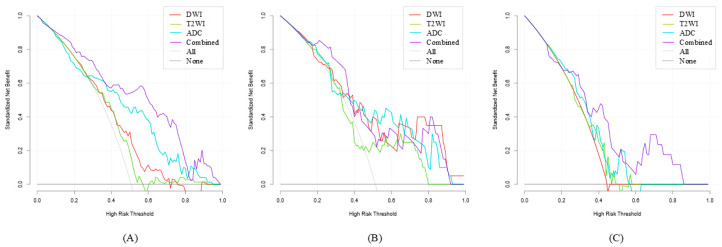
DCA curves for the Ridge model across the three cohorts. (**A**) The training cohort. (**B**) The validation cohort. (**C**) The test cohort.

The calibration curve for the Ridge model is presented in Figure 7, demonstrating the agreement between predicted probabilities and observed outcomes. Furthermore, DCA indicated that the Ridge outperformed both the “no assessment” and “comprehensive assessment” strategies, with threshold probability ranges of 10% to 92% and 21% to 86% in the validation and test cohorts, respectively. Detailed DCA curves are presented in Figure 8. Calibration curves and DCA curves for the remaining models are provided in Supplementary Figures S3 and S4.

### 3.4. Lesion Distribution in TSR Groups

This study analyzed the anatomical distribution of lesions between the H-TSR and L-TSR groups, focusing on the peripheral zone, transition zone, and cross-zone (involving both zones). Across the two centers, 55 lesions were located in the peripheral zone, 59 in the transition zone, and 109 in the cross-zone. In the H-TSR group, lesions were distributed as 32 (27.8%) in the peripheral zone, 35 (30.4%) in the transition zone, and 48 (41.8%) in the cross-zone. In the L-TSR group, the corresponding distributions were 23 (21.3%), 24 (22.2%), and 61 (56.5%), respectively. Chi-square testing revealed no statistically significant differences in lesion distribution between the H-TSR and L-TSR groups (*p* = 0.088). These findings are summarized in Table 3.

## 4. Discussion

The growth and biological behavior of tumors are profoundly regulated by the tumor stroma. Emerging evidence indicates that stromal secreted transforming growth factor-β (TGF-β) and modulated WNT signaling pathways play pivotal roles in the epithelial-stromal interactions of PCa [23,24,25]. In this study, five binary classification ML models were developed based on radiomic features to predict the TSR in PCa patients. The Ridge classifier demonstrated superior diagnostic performance, achieving robust and consistent predictive accuracy in both internal and external validation cohorts (AUC: 0.789 vs. 0.745; accuracy: 0.72 vs. 0.68), underscoring its clinical reliability for TSR stratification.

A total of 1746 radiomic features were initially extracted, with 28 significant features ultimately identified for model construction. Notably, majority of these features (89.29%) were derived from apparent ADC and DWI sequences. This observation aligns with findings by Cai et al. [14], who reported a similar predominance of ADC/DWI-derived features (62.50%) in TSR prediction models for rectal cancer. Current consensus posits that low-stroma PCa exhibits densely packed tumor cells with reduced extracellular space, leading to more pronounced restriction of water molecule diffusion [14,26]. Given the inherent sensitivity of DWI and ADC maps to microenvironmental diffusion properties, these sequences effectively capture cellular density and stromal architectural alterations, thereby serving as optimal sequences for TSR prediction.

Further feature analysis revealed that texture features accounted for the largest proportion (82.14%) of all identified features. This finding aligns with prior studies in pancreatic ductal adenocarcinoma [22,27,28], where textural features constituted the highest proportion (58.33–75%). Texture features not only characterize tumor complexity and heterogeneity by quantifying pixel intensity distributions but also capture subtle structural variations within pathological tissues through spatial relationships and local intensity changes [29,30]. Specifically, cancer-associated fibroblasts (CAFs), a predominant stromal component, mediate extracellular matrix (ECM) deposition and remodeling by secreting collagen, fibronectin, and periostin [31]. This dynamic process fosters heterogeneous fibrillar networks, increasing architectural complexity while inducing density variations and disorganized collagen fiber alignment [7,32]. Furthermore, ECM remodeling and increased localized fibrosis contribute to the formation of hypoxic regions, thereby activating hypoxia-inducible factor-1 (HIF-1), which regulates tumor-associated angiogenesis, leading to the proliferation of disorganized and functionally abnormal neovasculature [33]. Hypoxia also drives tumor metabolic reprogramming, including enhanced glycolysis and lactate accumulation [34], collectively amplifying tumor heterogeneity and metabolic dysregulation. Additionally, immune cells are recruited into the tumor stroma, where they secrete cytokines. For instance, tumor-associated macrophages (TAMs) release interleukin-10 (IL-10), which suppresses cytotoxic T-cell antitumor responses [35]. This immunosuppressive microenvironment, in synergy with ECM remodeling, angiogenesis, and metabolic alterations, collectively contributes to the complexity and heterogeneity of the tumor stroma. These dynamic changes exhibit distinct spatial variations between stroma-rich and stroma-poor tumors, which are precisely captured and reflected by texture features.

Furthermore, the top three radiomic features identified by SHAP analysis—DWI_log-sigma-1-mm-3D_glszm_ZonePercentage, ADC_wavelet-LHH_glcm_Correlation, and ADC_log-sigma-4-mm-3D_ngtdm_Busyness—were all derived from DWI and ADC and exclusively belonged to texture feature ZonePercentage, a GLSZM feature extracted from DWI, quantifies the proportion of small-scale homogeneous zones within a lesion [36]. This feature exhibited markedly lower values in the H-TSR group, indicating a greater number of fragmented, yet uniform, regions—suggestive of increased microstructural complexity associated with dense tumor cell aggregation. In contrast, Correlation and Busyness—calculated respectively from wavelet-transformed ADC using GLCM and from log-filtered ADC using NGTDM—reflect the degree of gray-level linear dependency and local intensity variation [37]. In lesions with abundant stromal content, Correlation values were consistently reduced, while Busyness was elevated, suggesting more disorganized architectural patterns and pronounced intratumoral heterogeneity. These findings underscore the biological plausibility of these features in characterizing tumor–stroma interactions and highlight their critical role in enhancing the interpretability and robustness of the model.

In this study, we evaluated five supervised learning models commonly employed for binary classification tasks: LR, SVM, BernoulliNBBayes, Ridge, and SGD. LR and BernoulliNBBayes were chosen as interpretable baseline models, Ridge was employed as a robust linear model with strong regularization, while SVM and SGD represented more sophisticated models capable of handling linear and nonlinear data distributions, respectively [38,39]. By encompassing a diverse range of data distribution characteristics and model complexities, these five models collectively provide a comprehensive evaluation framework, facilitating the identification of the most suitable classifier tailored to the specific nature of our dataset. The Ridge demonstrated superior predictive performance, achieving an AUC of 0.789 in the validation cohort and 0.745 in the external test cohort, followed by LR (validation AUC = 0.797; test AUC = 0.658). This outcome may be attributed to the linear distribution characteristics of our dataset. Prior studies [39,40] have established that linear models effectively capture underlying data patterns in small sample sizes, demonstrating strong generalization ability. The enhanced performance of Ridge regression is mechanistically linked to its L2 regularization framework. By introducing a penalty term proportional to the squared sum of model parameters into the loss function, L2 regularization effectively suppresses excessive weight fluctuations, thereby improving model robustness and stability—a particularly beneficial attribute in studies with limited sample sizes (*n* = 223) [39]. Similarly, Zhong et al. [41] reported that Ridge outperformed other models in predicting tumor hypoxia status prior to radiotherapy in PCa, further underscores its utility in assessing tumor microenvironmental alterations. Notably, different results were observed in a breast cancer mortality prediction study by Zhen et al. [42], where RF outperformed Ridge in both accuracy and stability. This discrepancy likely stems from their substantially larger sample size (1390 patients), which provided sufficient data dimensionality to leverage RF’s inherent strengths in handling high-dimensional, nonlinear relationships while avoiding overfitting [43]. While Ridge may not consistently outperform all other models, its optimal performance in this study underscores its specific advantages when aligned with specific experimental objectives and dataset characteristics. These results emphasize the interplay among algorithmic properties, biological question complexity, and available data resources. It is important to acknowledge that the sample sizes of the validation (*n* = 39) and test (*n* = 38) cohorts were relatively small. Although the Ridge demonstrated slightly superior performance, the limited sample size may have impaired the ability to detect subtle differences between models through the DeLong test (*p* > 0.05). The IDI provides a more sensitive evaluation, as even subtle probabilistic differences can be captured, despite modest overall AUC differences, thereby more effectively highlighting the superior performance of the Ridge in predicting PCa TSR.

The GS is the most widely applied and clinically relevant pathological grading system for PCa, providing a direct assessment of tumor cell differentiation. However, its evaluation is restricted to tumor cell morphology and our study highlights the stromal component within the TME, which may offer additional prognostic value. Therefore, incorporating radiomics-based TSR prediction into the GS grading system may facilitate more precise patient stratification. For instance, among patients with an identical GS grade, those with low TSR (stroma-rich tumors) may exhibit greater invasive potential and therefore require more aggressive management strategies. Conversely, patients with a high GS but concomitantly high TSR may suggest that, despite poor cellular differentiation, the presence of a relatively robust stromal barrier could mitigate aggressiveness, thereby helping to avoid overtreatment. Particularly in borderline cases such as GS 3 + 4 versus 4 + 3, imaging-derived TSR has the potential to provide valuable complementary information to guide clinical decision-making between active intervention and surveillance. Moreover, the DCA results indicated that all five models demonstrated substantial clinical net benefit across a wide range of threshold probabilities, with the Ridge showing particularly outstanding performance. However, translating these findings into clinical practice requires consideration of how such models may complement existing risk stratification systems. The National Comprehensive Cancer Network (NCCN) risk stratification system for PCa classifies patients based on PSA levels, GS, and clinical stage [44]. By integrating the DCA results of our model with the NCCN risk stratification system could further enable more comprehensive and individualized risk evaluation for patients. In addition, the model’s performance at lower DCA thresholds (validation cohort < 10% and test cohort < 21%) may enhance sensitivity, but at the cost of potentially overtreating low-risk patients. Conversely, higher thresholds (validation cohort > 92% and test cohort > 86%) can reduce false positives but may risk missing high-risk cases. When combined with NCCN risk stratification, threshold selection can be adaptively adjusted based on the patient’s baseline risk level [45]. For high-risk individuals, more lenient thresholds may be appropriate to maximize sensitivity and ensure timely identification of aggressive disease. For low-risk patients, stricter thresholds can help avoid unnecessary interventions. Such an approach may optimize the clinical utility of the model across heterogeneous patient populations.

The present study revealed no significant differences in lesion distribution between the H-TSR and L-TSR groups across the PZ and TZ of the prostate. This finding provides novel insights into the role of TSR across distinct anatomical regions. Previous research [46] has established that TZ-originated PCa generally demonstrate lower biological aggressiveness and more favorable clinical outcomes, whereas PZ tumors exhibit greater invasive potential and poorer prognosis. However, our results indicate that despite these established zonal differences in tumor biology, the distribution patterns of H-TSR and L-TSR lesions show no significant regional predilection. This suggests that TSR may represent a feature independent of anatomical zonation, with its homogeneous distribution across prostatic regions potentially reflecting underlying biological mechanisms governing tumor progression. One possible explanation is that PCa lesions across different anatomical regions are collectively influenced by global molecular regulatory networks [47]. These networks may include driver gene mutations [48,49], activation of key signaling pathways [47,50], and dynamic remodeling of the tumor microenvironment [51], all of which likely play a dominant role in shaping tumor behavior and characteristics. These global mechanisms transcend anatomical constraints, leading to consistent biological traits across different prostate regions. From a translational perspective, the zonal independence of TSR distribution carries significant implications for radiomics analysis. The observed spatial uniformity enables the development of imaging biomarkers that circumvent zonal segmentation requirements, thereby enhancing model generalizability and scalability across diverse clinical scenarios.

This study has several limitations. First, the retrospective design inherently introduces potential selection bias in case enrollment, compounded by the relatively small sample sizes from both participating centers. In particular, the validation and test cohorts consisted of only 39 and 38 patients, respectively, and such limited sample sizes are insufficient to substantiate reliable conclusions about the robustness and generalizability of the ML models. Therefore, large-scale multicenter prospective studies are warranted to validate the generalizability of our proposed model. Second, lesion delineation relied on manual segmentation rather than automated processing. However, a recent comparative study [52] demonstrated that current computer-aided PCa detection systems generate substantially higher false-positive rates compared to radiologists’ assessments. Therefore, the suitability of automated detection systems for clinical practice requires further investigation. Third, our model was developed based solely on imaging data, without incorporating clinical variables, genomic information, or other multi-omics data. In future studies, we aim to incorporate automated feature selection and multimodal data fusion in larger, multicenter cohorts to address the imbalance between high-dimensional features and limited sample sizes, thereby improving the robustness and generalizability of the model. Moreover, although multiple models were compared in this study, the small sample size may have limited the statistical power, and these conclusions require further validation in larger, multicenter datasets.

Despite the aforementioned limitations, this study represents the first exploratory investigation employing a bpMRI-based radiomics framework for the noninvasive prediction of TSR in PCa, highlighting its potential as a valuable tool for pathological characterization. Among the five ML models, the Ridge exhibited the best performance, with AUCs of 0.789 and 0.745 in the internal validation and external test cohorts, respectively. These findings provide preliminary evidence supporting the robustness and generalizability of the model, although confirmation in larger, multicenter cohorts remains essential. Notably, most of the selected features were texture-based metrics derived from DWI and ADC maps, effectively capturing the microstructural complexity and spatial heterogeneity of the TME. Additionally, TSR distribution did not differ significantly between the PZ and TZ, indicating that its prognostic value is independent of tumor anatomical location and reinforcing its potential as a broadly applicable imaging biomarker. Collectively, this exploratory study offers hypothesis-generating insights that may guide the development of personalized treatment strategies and provide novel perspectives for PCa management.

## Figures and Tables

**Table 1:** Demographic and clinical characteristics of the study patients across the three cohorts.

Characteristics	Training Cohort		*p*-Value	Testing Cohort		*p*-Value	Validation Cohort		*p*-Value
	H-TSR	L-TSR		H-TSR	L-TSR		H-TSR	L-TSR	
	146			39			38		<0.001 *
Case, n (%)	74 (50.68)	72 (49.32)	0.907	20 (51.28)	19 (48.72)	1.000	21 (55.26)	17 (33.74)	0.491
Age, mean ± SD, year	72.43 ± 8.24			74.38 ± 6.69			72.86 ± 9.21		0.063
	72.31 ± 7.76	73.56 ± 8.14	0.858	71.55 ± 6.61	77.37 ± 5.48	0.005 *	73.12 ± 8.57	69.67 ± 10.25	0.540
PSA, M(Q), ng/mL	35.30 (11.98, 100.00)			53.80 (13.3, 96.9)			5.00 (3.00, 75.40)		<0.001 *
	24.60 (11, 100.00)	41.15 (13.30, 100.00)	0.129	19.05 (10.10, 64.35)	85.50 (36.50, 100.00)	0.003 *	11.20 (3.00, 100)	5.00 (4.25, 12.24)	0.946
fPAS, M(Q), ng/mL	3.12 (1.21, 12.73)			4.70 (1.74, 12.1)			8.85 (3.08, 30.00)		0.006 *
	2.70 (0.89, 8.40)	3.44 (1.32, 23.45)	0.042 *	1.95 (1.46, 5.20)	6.86 (4.42, 22.90)	0.003 *	8.85 (1.87, 30.00)	18.00 (4.79, 30.00)	0.318
fPSA/PSA, M(Q)	0.12 (0.08, 0.24)			0.12 (0.07, 0.23)			1.06 (0.16, 5.69)		<0.001 *
	0.12 (0.07, 0.18)	0.13 (0.09, 0.25)	0.099	0.12 (0.07, 0.20)	0.13 (0.07, 0.25)	0.588	0.3 (0.15, 2.55)	1.16 (0.18, 6.00)	0.373
Prostate volume, M(Q), cm^3^	57.64 (41.13, 81.41)			56.39 (46.74, 74.86)			38.95 (26.63, 55.29)		<0.001 *
	58.03 (41.02, 78.33)	56.79 (41.71, 87.69)	0.899	54.63 (43.57, 69.75)	56.39 (48.86, 78.02)	0.235	51.2 (29.52, 64.37)	34.03 (22.57, 39.81)	0.028 *
PSAD, M(Q), ng/mL^2^	0.59 (0.23, 1.31)			0.78 (0.24, 1.53)			0.14 (0.05, 0.99)		0.011 *
	0.46 (0.18, 1.20)	0.77 (0.27, 1.35)	0.095	0.38 (0.15, 1.19)	1.04 (0.69, 1.60)	0.021 *	0.31 (0.47, 1.53)	0.14 (0.13, 0.54)	0.876
PI-RADS score, n (%)									0.171
			0.052			0.438			0.517
1 or 2	0 (0.00)	0 (0.00)		0 (0.00)	1 (5.26)		0 (0.00)	0 (0.00)	
3	12 (16.22)	4 (5.56)		1 (5.00)	3 (15.79)		2 (9.52)	3 (17.65)	
4	4 (5.41)	9 (12.50)		3 (15.00)	4 (21.05)		3 (14.29)	4 (23.53)	
5	58 (78.38)	59 (81.94)		16 (80.00)	12 (63.16)		16 (74.19)	10 (58.82)	

fPSA, free PSA; M, medians; PSA, prostate-specific antigen; PSAD, PSA density; Q, 25th and 75th percentiles; SD, standard deviation. * Indicates statistical significance at *p* < 0.05.

**Table 2:** Performance evaluation of the Ridge model across the three cohorts.

Cohorts	AUC (95% CI)	Accuracy (95% CI)	Specificity (95% CI)	Recall (95% CI)	F1-Score	PPV	NPV
Training	0.846 (0.782–0.909)	0.80 (0.796–0.807)	0.86 (0.852–0.871)	0.74 (0.732–0.755)	0.79	0.8	0.77
Validation	0.789 (0.648–0.931)	0.72 (0.695–0.741)	0.47 (0.422–0.525)	0.95 (0.929–0.971)	0.78	0.66	0.90
Test	0.745 (0.583–0.907)	0.68 (0.660–0.708)	0.67 (0.623–0.711)	0.71 (0.653–0.758)	0.67	0.63	0.74

CI, confidence interval; NPV, negative predictive value.

**Table 3:** Lesion distribution proportions in the H-TSR and the L-TSR groups: transition zone and peripheral zone.

TSR	Peripheral Zone (*n*, %)	Transition Zone (*n*, %)	Cross-Zone (*n*, %)	Total	*p*
H-TSR	32 (27.8%)	35 (30.4%)	48 (41.8%)	115	0.088
L-TSR	23 (21.3%)	24 (22.2%)	61 (56.5%)	108	
Total	55 (24.5%)	59 (26.5%)	109 (49.0%)	223	

## Data Availability

The data presented in this study are available on request from the corresponding author. The data are not publicly available due to ethical restrictions.

## Supplementary Materials

The following supporting information can be downloaded at: https://www.mdpi.com/article/10.3390/diagnostics15212722/s1, Figure S1: Calibration curves demonstrating the performance of the LR, SVM, BernoulliNB, and SGD models in the internal validation, external validation, and combined cohorts; Figure S2: Calibration curves of the LR, SVM, BernoulliNB, and SGD models in the internal validation, external validation, and combined cohorts; Table S1: MRI acquisition parameters; Table S2: Binary Logistic Regression Analysis of Clinical Variables; Table S3: AUC performance of five ML models using single and combined MRI sequences for TSR prediction in PCa; Table S4: Performance evaluation of the five ML models across the three cohorts; Table S5: DeLong test results for ROC curves of different cohorts; Table S6: Comparison of IDI between Ridge and other models across different datasets.
