# Supplementary material for: Advancing Prostate Cancer Assessment: A Biparametric MRI (T2WI and DWI/ADC)-Based Radiomic Approach to Predict Tumor–Stroma Ratio
Source: Diagnostics (Basel). 2025 Oct 27;15(21):2722. doi: 10.3390/diagnostics15212722 (PMC12609615; doi:10.3390/diagnostics15212722)
Supplement: Supplementary file 1 [file diagnostics-15-02722-s001.zip › Non-published Material/Supplementary Tables.docx]

**Supplementary Table 1.** MRI acquisition parameters.

|  | **Image** | **TE (ms)** | **TR (ms)** | **Slice Thickness(mm)** | **FOV read (mm)/FOV Phase %** | **FOV (mm)** | **Acquisition Matrix** | **b values(s/mm^2)** |
| --- | --- | --- | --- | --- | --- | --- | --- | --- |
| **MAGNETOM Prisma** | (FS)-T2WI | 85 | 3200 | 3.5 | 200/100 | 200 × 200 | 320 × 224 | - |
|  | DWI | 78 | 3700 | 3.5 | 200/100 | 200 × 200 | 140 × 140 | 0, 50，1000，1400 |
|  |  |  |  |  |  |  |  |  |
| **MAGNETOM Verio** | (FS)-T2WI | 77 | 3090 | 3 | 320/70 | 200 × 200 | 320 × 240 | - |
|  | DWI | 84 | 3800 | 3 | 320/70 | 200 × 200 | 118 × 118 | 0, 50，1000，1400，2000 |

TR, repetition time; TE, echo time; FOV, field of view; FS, fat suppression.

**Supplementary Table 2.** Binary Logistic Regression Analysis of Clinical Variables.

| Variable | Age | PSA | fPSA | fPSA/PSA | Prostate volume | PSAD | PI-RADS |
| --- | --- | --- | --- | --- | --- | --- | --- |
| *p* | 0.212 | 0.875 | 0.128 | 0.838 | 0.661 | 0.405 | 0.073 |

**Supplementary Table 3.** AUC performance of five ML models using single and combined MRI sequences for TSR prediction in PCa.

|  |  | **Logistic** | **SVM** | **Bernoulli-NBBayes** | **Ridge** | **SGD** |
| --- | --- | --- | --- | --- | --- | --- |
| **T2WI** | **Training** | 0.623 | 0.611 | 0.627 | 0.623 | 0.623 |
|  | **Validation** | 0.663 | 0.671 | 0.592 | 0.661 | 0.663 |
|  | **Test** | 0.420 | 0.417 | 0.606 | 0.412 | 0.420 |
| **DWI** | **Training** | 0.660 | 0.771 | 0.702 | 0.671 | 0.656 |
|  | **Validation** | 0.761 | 0.666 | 0.809 | 0.750 | 0.766 |
|  | **Test** | 0.541 | 0.518 | 0.585 | 0.546 | 0.541 |
| **ADC** | **Training** | 0.803 | 0.793 | 0.722 | 0.789 | 0.768 |
|  | **Validation** | 0.721 | 0.766 | 0.712 | 0.758 | 0.769 |
|  | **Test** | 0.546 | 0.605 | 0.636 | 0.686 | 0.636 |
| **T2WI+ DWI+ADC** | **Training** | 0.831 | 0.640 | 0.745 | 0.846 | 0.835 |
|  | **Validation** | 0.797 | 0.763 | 0.751 | 0.789 | 0.752 |
|  | **Test** | 0.658 | 0.755 | 0.672 | 0.745 | 0.695 |

**Supplementary Table 4.** Performance evaluation of the five ML models across the three cohorts.

| **Models** | **Cohorts** | **AUC (95% CI)** | **Accuracy (95% CI)** | **Specificity (95% CI)** | **Recall (95% CI)** | **F1-Score** | **PPV** | **NPV** |
| --- | --- | --- | --- | --- | --- | --- | --- | --- |
| **LR** | **Training** | 0.831 (0.765- 0.898) | 0.78 (0.775-0.786) | 0.86 (0.852-0.871) | 0.70 (0.691-0.715) | 0.76 | 0.84 | 0.74 |
|  | **Validation** | 0.797 (0.660-0.935) | 0.72 (0.695-0.741) | 0.53 (0.475-0.578) | 0.90 (0.871-0.929) | 0.77 | 0.67 | 0.83 |
|  | **Test** | 0.658 (0.478-0.839) | 0.66 (0.633-0.682) | 0.67 (0.623-0.711) | 0.65 (0.592-0.702) | 0.63 | 0.61 | 0.70 |
|  |  |  |  |  |  |  |  |  |
| **SVM** | **Training** | 0.640 (0.548-0.732) | 0.64 (0.637-0.65) | 0.60 (0.584-0.611) | 0.69 (0.677-0.701) | 0.66 | 0.64 | 0.67 |
|  | **Validation** | 0.763 (0.610-0.917) | 0.72 (0.695-0.741) | 0.89 (0.863-0.926) | 0.55 (0.501-0.599) | 0.67 | 0.85 | 0.65 |
|  | **Test** | 0.755 (0.594-0.916) | 0.74 (0.714-0.76) | 0.67 (0.623-0.711) | 0.82 (0.780-0.867) | 0.74 | 0.67 | 0.82 |
|  |  |  |  |  |  |  |  |  |
| **Bernoulli-NBBayes** | **Training** | 0.745 (0.665-0.824) | 0.70 (0.692-0.705) | 0.72 (0.710-0.734) | 0.68 (0.663-0.688) | 0.69 | 0.71 | 0.68 |
|  | **Validation** | 0.751 (0.598-0.905) | 0.69 (0.669-0.716) | 0.84 (0.804-0.880) | 0.55 (0.501-0.599) | 0.65 | 0.79 | 0.64 |
|  | **Test** | 0.672 (0.492-0.852) | 0.63 (0.607-0.656) | 0.62 (0.574-0.664) | 0.65 (0.592-0.702) | 0.61 | 0.58 | 0.67 |
|  |  |  |  |  |  |  |  |  |
| **Ridge** | **Training** | 0.846 (0.782-0.909) | 0.80 (0.796-0.807) | 0.86 (0.852-0.871) | 0.74 (0.732-0.755) | 0.79 | 0.8 | 0.77 |
|  | **Validation** | 0.789 (0.648-0.931) | 0.72 (0.695-0.741) | 0.47 (0.422-0.525) | 0.95 (0.929-0.971) | 0.78 | 0.66 | 0.90 |
|  | **Test** | 0.745 (0.583-0.907) | 0.68 (0.660-0.708) | 0.67 (0.623-0.711) | 0.71 (0.653-0.758) | 0.67 | 0.63 | 0.74 |
|  |  |  |  |  |  |  |  |  |
| **SGD** | **Training** | 0.835 (0.769-0.901) | 0.77 (0.768-0.780) | 0.82 (0.809-0.830) | 0.73 (0.718-0.741) | 0.77 | 0.81 | 0.75 |
|  | **Validation** | 0.753 (0.599-0.906) | 0.67 (0.643-0.690) | 1.00 (1.0-1.0) | 0.35 (0.303-0.397) | 0.52 | 1.00 | 0.59 |
|  | **Test** | 0.695 (0.522-0.867) | 0.68 (0.660-0.708) | 0.76 (0.722-0.802) | 0.59 (0.531-0.645) | 0.63 | 0.67 | 0.70 |

CI, confidence interval; NPV, negative predictive value.

**Supplementary Table 5.** DeLong test results for ROC curves of different cohorts.

|  | **Variables** | **LR** | **SVM** | **BernoulliN-BBayes** | **Ridge** | **SGD** |
| --- | --- | --- | --- | --- | --- | --- |
| **Training** | **LR** | 1.000 | ＜0.05* | 0.014* | 0.356 | 0.707 |
|  | **SVM** | ＜0.05* | 1.000 | 0.059 | ＜0.05* | ＜0.05* |
|  | **BernoulliNBBayes** | 0.014* | 0.059 | 1.000 | 0.005* | 0.026* |
|  | **Ridge** | 0.356 | ＜0.05* | 0.005* | 1.000 | 0.573 |
|  | **SGD** | 0.707 | ＜0.05* | 0.026* | 0.573 | 1.000 |
| **Validation** | **LR** | 1.000 | 0.697 | 0.534 | 0.751 | 0.105 |
|  | **SVM** | 0.697 | 1.000 | 0.899 | 0.775 | 0.912 |
|  | **BernoulliNBBayes** | 0.534 | 0.899 | 1.000 | 0.627 | 0.989 |
|  | **Ridge** | 0.751 | 0.775 | 0.627 | 1.000 | 0.278 |
|  | **SGD** | 0.105 | 0.912 | 0.989 | 0.278 | 1.000 |
| **Test** | **LR** | 1.000 | 0.254 | 0.893 | 0.340 | 0.241 |
|  | **SVM** | 0.254 | 1.000 | 0.480 | 0.920 | 0.457 |
|  | **BernoulliNBBayes** | 0.893 | 0.480 | 1.000 | 0.595 | 0.844 |
|  | **Ridge** | 0.340 | 0.920 | 0.595 | 1.000 | 0.484 |
|  | **SGD** | 0.241 | 0.457 | 0.844 | 0.484 | 1.000 |

* Difference was significant at the given level.

**Supplementary Table 6**. Comparison of IDI between Ridge and other models across different datasets.

| **Comparison models** | **IDI** | | |
| --- | --- | --- | --- |
|  | **Training set** | **Validation set** | **Test set** |
| **Ridge vs LR** | 0.04 | 0.01 | 0.06 |
| **Ridge vs SVM** | 0.31 | 0.02 | -0.10 |
| **Ridge vs BernoulliNBBayes** | 0.20 | 0.03 | 0.08 |
| **Ridge vs SGD** | 0.05 | 0.07 | 0.03 |

IDI, integrated discrimination improvement.
