# Supplementary material for: Advancing Prostate Cancer Assessment: A Biparametric MRI (T2WI and DWI/ADC)-Based Radiomic Approach to Predict Tumor–Stroma Ratio
Source: Diagnostics (Basel). 2025 Oct 27;15(21):2722. doi: 10.3390/diagnostics15212722 (PMC12609615; doi:10.3390/diagnostics15212722)
Supplement: Supplementary file 1 [file diagnostics-15-02722-s001.zip › Non-published Material/Supplementary material.docx]

Detailed Description of Image Preprocessing, Feature Extraction, and Model Parameter Settings：

**1. Image Preprocessing Workflow**

To ensure consistency and reproducibility of multicenter imaging data, all MRI scans underwent standardized preprocessing prior to analysis, as outlined below:

**Co-registration**

- DWI/ADC images were automatically co-registered to T2WI using ITK-SNAP software (v4.0.1).
- This step ensured that all features were extracted within a unified spatial reference, avoiding errors from inter-sequence misalignment.

**Bias field correction**

- Intensity non-uniformities were corrected using the N4ITK algorithm.
- This reduced low-frequency intensity artifacts caused by magnetic field inhomogeneity.

**Intensity normalization**

- Z-score normalization was applied to all images, rescaling intensity values to the [0,1] range.
- This reduced variability across patients and scanning batches, improving feature stability.

**Spatial resampling**

- Images were resampled to an isotropic voxel size of 1.0 × 1.0 × 1.0 mm³ using B-spline interpolation.
- This guaranteed spatial consistency across patients, facilitating reliable cross-comparisons of radiomic features.

**Filtering and enhancement**

- Wavelet transforms were applied prior to feature extraction.
- These enhanced edge and texture information, ensuring robust capture of both high- and low-frequency patterns.

**Quality control**

- All ROIs and preprocessing results were independently reviewed by two radiologists with >8 and >12 years of prostate MRI experience.
- Discrepancies were resolved by consensus discussion, ensuring reliable tumor boundary delineation.

**2. ROI Delineation and Radiomic Feature Extraction**

**ROI delineation criteria**

- ROIs encompassed the solid portion of the tumor (whole tumor volume), excluding necrotic, cystic, or edematous regions.
- T2WI served as the primary reference sequence, supplemented by high-signal regions on high–b value DWI (b = 1400 s/mm²) and corresponding hypointense regions on ADC maps.
- DWI/ADC images were automatically co-registered to T2WI in ITK-SNAP.

**Delineation process**

- ROIs were manually delineated in three dimensions by a radiologist with >8 years of prostate MRI experience.
- The delineations were independently reviewed by a second radiologist with >15 years of experience.
- In cases of disagreement, consensus was reached through discussion.

**Feature extraction**

- Radiomic features were extracted using the InferScholar platform (v6.0, InferVision, Beijing, China).
- Feature categories included:
  - First-order statistical features
  - Shape-based features
  - Texture features: GLCM, GLRLM, GLSZM, NGTDM, GLDM
  - Wavelet-transformed features
- A total of **1,746 features** were initially extracted for each lesion.

**3. Feature Selection and Dimensionality Reduction**

**Reproducibility assessment**

- Inter-observer reproducibility was assessed by calculating ICC values.
- Features with ICC > 0.80 were retained for further analysis.

**Redundancy elimination**

- Spearman correlation analysis was applied, and features with absolute correlation coefficients >0.85 were removed.

**Feature selection**

- LASSO regression was then used for dimensionality reduction, resulting in a final set of **28 stable and informative features**.

**4. Model Development and Hyperparameter Optimization**

**Machine learning classifiers**
Five widely used machine learning classifiers were developed in this study:

- Logistic Regression (**LR**)
- Support Vector Machine (**SVM**)
- BernoulliNaïveBayes
- Ridge Classifier (**Ridge**)
- Stochastic Gradient Descent Classifier (**SGD**)

**Hyperparameter tuning strategy**

- **Method:** Five-fold cross-validation (5-fold CV).
- **Evaluation metric:** Mean AUC across folds.
- **Search approach:** Tree-structured Parzen Estimator (TPE) Bayesian optimization using the *hyperopt* library.
- **Maximum iterations:** 100 search trials.
- **Final configuration:** The hyperparameter set yielding the highest mean cross-validated AUC was selected as the optimal configuration for each model.

**Search spaces (Python code)**

# Support Vector Machine (SVM)

space = {

'kernel': hp.choice('kernel', ['linear', 'rbf', 'poly', 'sigmoid']),

'C': hp.loguniform("C", np.log(0.01), np.log(100)),

'gamma': hp.loguniform("gamma", np.log(0.001), np.log(0.1)),

'degree': hp.loguniform("degree", np.log(0.01), np.log(10)),

'coef0': hp.loguniform("coef0", np.log(0.01), np.log(10))

}

# BernoulliNaïveBayes

space = {

'alpha': hp.loguniform("alpha", np.log(0.01), np.log(100)),

'binarize': hp.loguniform("binarize", np.log(0.001), np.log(10))

}

# Ridge Classifier (Ridge)

space = {

'alpha': hp.loguniform("alpha", np.log(0.01), np.log(100)),

'binarize': hp.loguniform("binarize", np.log(0.001), np.log(10))

}

# Stochastic Gradient Descent Classifier (SGD)

space = {

'alpha': hp.loguniform("alpha", np.log(0.01), np.log(100))

}

# Logistic Regression (LR, SGD-based)

space = {

'loss': hp.choice('loss', ['log']),

'penalty': hp.choice('penalty', ['l2', 'l1', 'elasticnet']),

'alpha': hp.loguniform('alpha', np.log(0.01), np.log(100))

}
