# Supplementary material for: Advancing Prostate Cancer Assessment: A Biparametric MRI (T2WI and DWI/ADC)-Based Radiomic Approach to Predict Tumor–Stroma Ratio
Source: Diagnostics (Basel). 2025 Oct 27;15(21):2722. doi: 10.3390/diagnostics15212722 (PMC12609615; doi:10.3390/diagnostics15212722)
Supplement: Supplementary file 1 [file diagnostics-15-02722-s001.zip › Supplementary material.pdf]

Detailed Description of Image Preprocessing, Feature Extraction, and Model Parameter Settings：

1. Image Preprocessing Workflow

To ensure consistency and reproducibility of multicenter imaging data, all MRI scans underwent standardized preprocessing prior to analysis, as outlined below:

Co-registration

- DWI/ADC images were automatically co-registered to T2WI using ITK-SNAP software (v4.0.1).
- This step ensured that all features were extracted within a unified spatial reference, avoiding errors from inter-sequence misalignment.

Bias field correction

- Intensity non-uniformities were corrected using the N4ITK algorithm.
- This reduced low-frequency intensity artifacts caused by magnetic field inhomogeneity.

Intensity normalization

- Z-score normalization was applied to all images, rescaling intensity values to the [0,1] range.
- This reduced variability across patients and scanning batches, improving feature stability.

Spatial resampling

- Images were resampled to an isotropic voxel size of 1.0 × 1.0 × 1.0 mm^3 using B-spline interpolation.
- This guaranteed spatial consistency across patients, facilitating reliable cross-comparisons of radiomic features.

Filtering and enhancement

- Wavelet transforms were applied prior to feature extraction.
- These enhanced edge and texture information, ensuring robust capture of both high- and low-frequency patterns.

Quality control

- All ROIs and preprocessing results were independently reviewed by two radiologists with >8 and >12 years of prostate MRI experience.
- Discrepancies were resolved by consensus discussion, ensuring reliable tumor boundary delineation.

---

2. ROI Delineation and Radiomic Feature Extraction

ROI delineation criteria

- ROIs encompassed the solid portion of the tumor (whole tumor volume), excluding necrotic, cystic, or edematous regions.
- T2WI served as the primary reference sequence, supplemented by high-signal regions on high–b value DWI (b = 1400 s/mm^2) and corresponding hypointense regions on ADC maps.
- DWI/ADC images were automatically co-registered to T2WI in ITK-SNAP.

Delineation process

- ROIs were manually delineated in three dimensions by a radiologist with >8 years of prostate MRI experience.
- The delineations were independently reviewed by a second radiologist with >15 years of experience.
- In cases of disagreement, consensus was reached through discussion.

Feature extraction

- Radiomic features were extracted using the InferScholar platform (v6.0, InferVision, Beijing, China).
- Feature categories included:
    - First-order statistical features
    - Shape-based features
    - Texture features: GLCM, GLRLM, GLSZM, NGTDM, GLDM
    - Wavelet-transformed features
- A total of **1,746 features** were initially extracted for each lesion.

3. Feature Selection and Dimensionality Reduction

Reproducibility assessment

- Inter-observer reproducibility was assessed by calculating ICC values.
- Features with ICC > 0.80 were retained for further analysis.

Redundancy elimination

- Spearman correlation analysis was applied, and features with absolute correlation coefficients >0.85 were removed.

Feature selection

- LASSO regression was then used for dimensionality reduction, resulting in a final set of **28 stable and informative features**.

4. Model Development and Hyperparameter Optimization

Machine learning classifiers

Five widely used machine learning classifiers were developed in this study:

- Logistic Regression (**LR**)
- Support Vector Machine (**SVM**)
- BernoulliNaïveBayes
- Ridge Classifier (**Ridge**)
- Stochastic Gradient Descent Classifier (**SGD**)

Hyperparameter tuning strategy

- **Method:** Five-fold cross-validation (5-fold CV).
- **Evaluation metric:** Mean AUC across folds.
- **Search approach:** Tree-structured Parzen Estimator (TPE) Bayesian optimization using the *hyperopt* library.
- **Maximum iterations:** 100 search trials.
- **Final configuration:** The hyperparameter set yielding the highest mean cross-validated AUC was selected as the optimal configuration for each model.

Search spaces (Python code)

```
# Support Vector Machine (SVM)
space = {
```

```
    'kernel': hp.choice('kernel', ['linear', 'rbf', 'poly', 'sigmoid']),
    'C': hp.loguniform("C", np.log(0.01), np.log(100)),
    'gamma': hp.loguniform("gamma", np.log(0.001), np.log(0.1)),
    'degree': hp.loguniform("degree", np.log(0.01), np.log(10)),
    'coef0': hp.loguniform("coef0", np.log(0.01), np.log(10))
}

# BernoulliNaïveBayes
space = {
    'alpha': hp.loguniform("alpha", np.log(0.01), np.log(100)),
    'binarize': hp.loguniform("binarize", np.log(0.001), np.log(10))
}

# Ridge Classifier (Ridge)
space = {
    'alpha': hp.loguniform("alpha", np.log(0.01), np.log(100)),
    'binarize': hp.loguniform("binarize", np.log(0.001), np.log(10))
}

# Stochastic Gradient Descent Classifier (SGD)
space = {
    'alpha': hp.loguniform("alpha", np.log(0.01), np.log(100))
}

# Logistic Regression (LR, SGD-based)
space = {
    'loss': hp.choice('loss', ['log']),
    'penalty': hp.choice('penalty', ['l2', 'l1', 'elasticnet']),
    'alpha': hp.loguniform('alpha', np.log(0.01), np.log(100))
}
```
