# Supplementary material for: Advancing Prostate Cancer Assessment: A Biparametric MRI (T2WI and DWI/ADC)-Based Radiomic Approach to Predict Tumor–Stroma Ratio
Source: Diagnostics (Basel). 2025 Oct 27;15(21):2722. doi: 10.3390/diagnostics15212722 (PMC12609615; doi:10.3390/diagnostics15212722)
Supplement: Supplementary file 1 [file diagnostics-15-02722-s001.zip › Supplementary Tables.pdf]

Supplementary Table 1. MRI acquisition parameters.

	Image	TE (ms)	TR (ms)	Slice Thickness(mm)	FOV read (mm)/FOV Phase %	FOV (mm)	Acquisition Matrix	b values(s/mm^2)
**MAGNETOM Prisma**	(FS)-T2WI	85	3200	3.5	200/100	200 × 200	320 × 224	-
	DWI	78	3700	3.5	200/100	200 × 200	140 × 140	0, 50，1000，1400
**MAGNETOM Verio**	(FS)-T2WI	77	3090	3	320/70	200 × 200	320 × 240	-
	DWI	84	3800	3	320/70	200 × 200	118 × 118	0, 50，1000，1400，2000

TR, repetition time; TE, echo time; FOV, field of view; FS, fat suppression.

Supplementary Table 2. Binary Logistic Regression Analysis of Clinical Variables.

Variable	Age	PSA	fPSA	fPSA/PSA	Prostate volume	PSAD	PI-RADS
p	0.212	0.875	0.128	0.838	0.661	0.405	0.073

Supplementary Table 3. AUC performance of five ML models using single and combined MRI sequences for TSR prediction in PCa.

		**Logistic**	**SVM**	**Bernoulli-NBBayes**	**Ridge**	**SGD**
**T2WI**	**Training**	0.623	0.611	0.627	0.623	0.623

	**Validation**	0.663	0.671	0.592	0.661	0.663
	**Test**	0.420	0.417	0.606	0.412	0.420
**DWI**	**Training**	0.660	0.771	0.702	0.671	0.656
	**Validation**	0.761	0.666	0.809	0.750	0.766
	**Test**	0.541	0.518	0.585	0.546	0.541
**ADC**	**Training**	0.803	0.793	0.722	0.789	0.768
	**Validation**	0.721	0.766	0.712	0.758	0.769
	**Test**	0.546	0.605	0.636	0.686	0.636
**T2WI+DWI+ADC**	**Training**	0.831	0.640	0.745	0.846	0.835
	**Validation**	0.797	0.763	0.751	0.789	0.752
	**Test**	0.658	0.755	0.672	0.745	0.695

Supplementary Table 4. Performance evaluation of the five ML models across the three cohorts.

Models	Cohorts	AUC (95% CI)	Accuracy (95% CI)	Specificity (95% CI)	Recall (95% CI)	F1-Score	PPV	NPV
LR	Training	0.831 (0.765-0.898)	0.78 (0.775-0.786)	0.86 (0.852-0.871)	0.70 (0.691-0.715)	0.76	0.84	0.74
	Validation	0.797 (0.660-0.935)	0.72 (0.695-0.741)	0.53 (0.475-0.578)	0.90 (0.871-0.929)	0.77	0.67	0.83
	Test	0.658 (0.478-0.839)	0.66 (0.633-0.682)	0.67 (0.623-0.711)	0.65 (0.592-0.702)	0.63	0.61	0.70
SVM	Training	0.640 (0.548-0.732)	0.64 (0.637-0.65)	0.60 (0.584-0.611)	0.69 (0.677-0.701)	0.66	0.64	0.67
	Validation	0.763 (0.610-0.917)	0.72 (0.695-0.741)	0.89 (0.863-0.926)	0.55 (0.501-0.599)	0.67	0.85	0.65
	Test	0.755 (0.594-0.916)	0.74 (0.714-0.76)	0.67 (0.623-0.711)	0.82 (0.780-0.867)	0.74	0.67	0.82
Bernoulli-NBBayes	Training	0.745 (0.665-0.824)	0.70 (0.692-0.705)	0.72 (0.710-0.734)	0.68 (0.663-0.688)	0.69	0.71	0.68
	Validation	0.751 (0.598-0.905)	0.69 (0.669-0.716)	0.84 (0.804-0.880)	0.55 (0.501-0.599)	0.65	0.79	0.64

	**Test**	0.672 (0.492-0.852)	0.63 (0.607-0.656)	0.62 (0.574-0.664)	0.65 (0.592-0.702)	0.61	0.58	0.67
**Ridge**	**Training**	0.846 (0.782-0.909)	0.80 (0.796-0.807)	0.86 (0.852-0.871)	0.74 (0.732-0.755)	0.79	0.8	0.77
	**Validation**	0.789 (0.648-0.931)	0.72 (0.695-0.741)	0.47 (0.422-0.525)	0.95 (0.929-0.971)	0.78	0.66	0.90
	**Test**	0.745 (0.583-0.907)	0.68 (0.660-0.708)	0.67 (0.623-0.711)	0.71 (0.653-0.758)	0.67	0.63	0.74
**SGD**	**Training**	0.835 (0.769-0.901)	0.77 (0.768-0.780)	0.82 (0.809-0.830)	0.73 (0.718-0.741)	0.77	0.81	0.75
	**Validation**	0.753 (0.599-0.906)	0.67 (0.643-0.690)	1.00 (1.0-1.0)	0.35 (0.303-0.397)	0.52	1.00	0.59
	**Test**	0.695 (0.522-0.867)	0.68 (0.660-0.708)	0.76 (0.722-0.802)	0.59 (0.531-0.645)	0.63	0.67	0.70

CI, confidence interval; NPV, negative predictive value.

Supplementary Table 5. DeLong test results for ROC curves of different cohorts.

	**Variables**	**LR**	**SVM**	**BernoulliN-BBayes**	**Ridge**	**SGD**
**Training**	**LR**	1.000	< 0.05*	0.014*	0.356	0.707
	**SVM**	< 0.05*	1.000	0.059	< 0.05*	< 0.05*
	**BernoulliNBBayes**	0.014*	0.059	1.000	0.005*	0.026*
	**Ridge**	0.356	< 0.05*	0.005*	1.000	0.573
	**SGD**	0.707	< 0.05*	0.026*	0.573	1.000
**Validation**	**LR**	1.000	0.697	0.534	0.751	0.105
	**SVM**	0.697	1.000	0.899	0.775	0.912
	**BernoulliNBBayes**	0.534	0.899	1.000	0.627	0.989
	**Ridge**	0.751	0.775	0.627	1.000	0.278
	**SGD**	0.105	0.912	0.989	0.278	1.000
**Test**	**LR**	1.000	0.254	0.893	0.340	0.241
	**SVM**	0.254	1.000	0.480	0.920	0.457
	**BernoulliNBBayes**	0.893	0.480	1.000	0.595	0.844
	**Ridge**	0.340	0.920	0.595	1.000	0.484
	**SGD**	0.241	0.457	0.844	0.484	1.000

* Difference was significant at the given level.

Supplementary Table 6. Comparison of IDI between Ridge and other models across different datasets.

Comparison models	IDI		
	Training set	Validation set	Test set
Ridge vs LR	0.04	0.01	0.06
Ridge vs SVM	0.31	0.02	-0.10
Ridge vs BernoulliNBBayes	0.20	0.03	0.08
Ridge vs SGD	0.05	0.07	0.03

IDI, integrated discrimination improvement.
