# Supplementary figures and images for: Advancing Prostate Cancer Assessment: A Biparametric MRI (T2WI and DWI/ADC)-Based Radiomic Approach to Predict Tumor–Stroma Ratio
Source: Diagnostics (Basel). 2025 Oct 27;15(21):2722. doi: 10.3390/diagnostics15212722 (PMC12609615; doi:10.3390/diagnostics15212722)

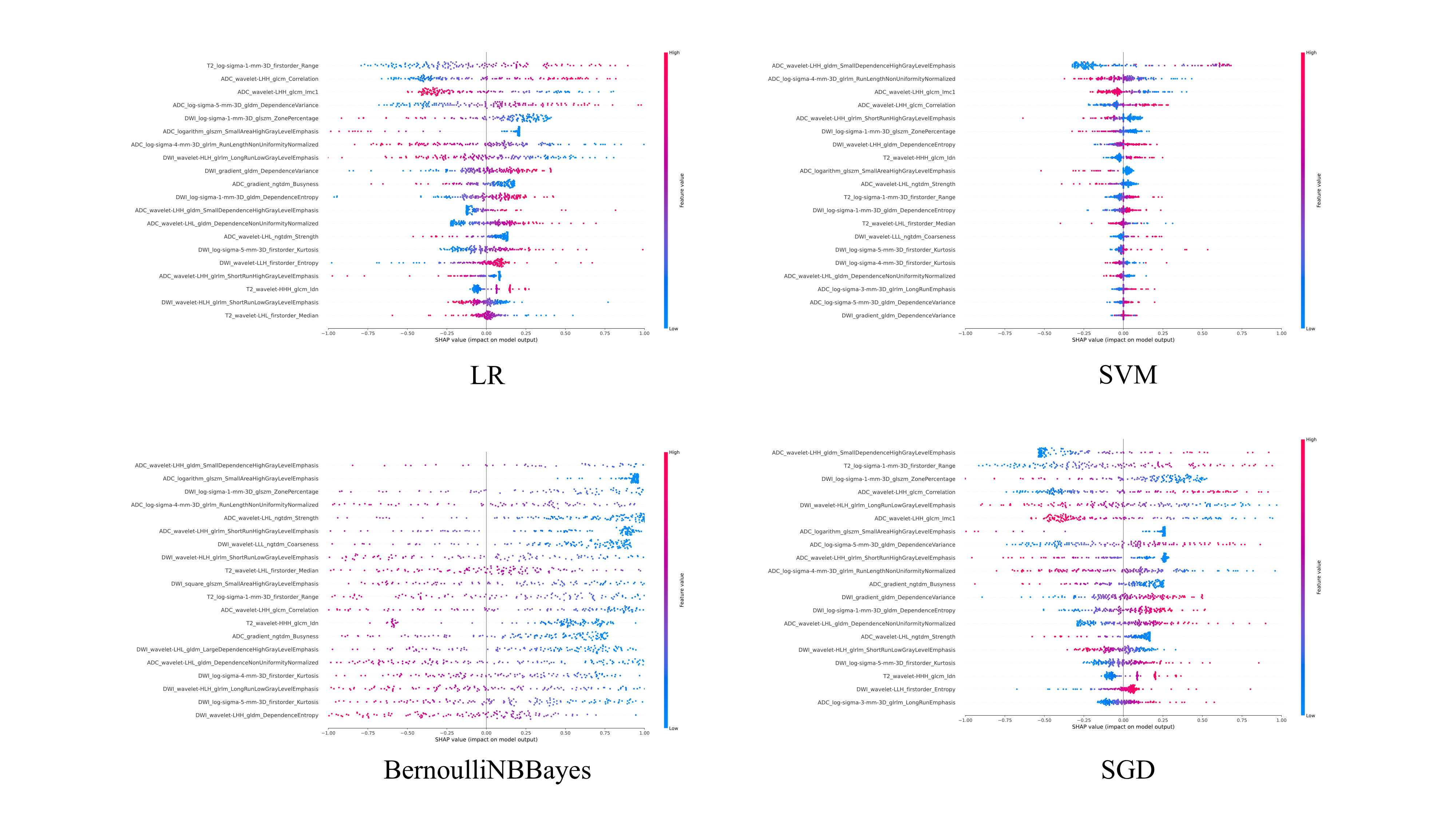

Supplement: Supplementary file 1 [file diagnostics-15-02722-s001.zip › Non-published Material/Supplementary Figure 1.tif]

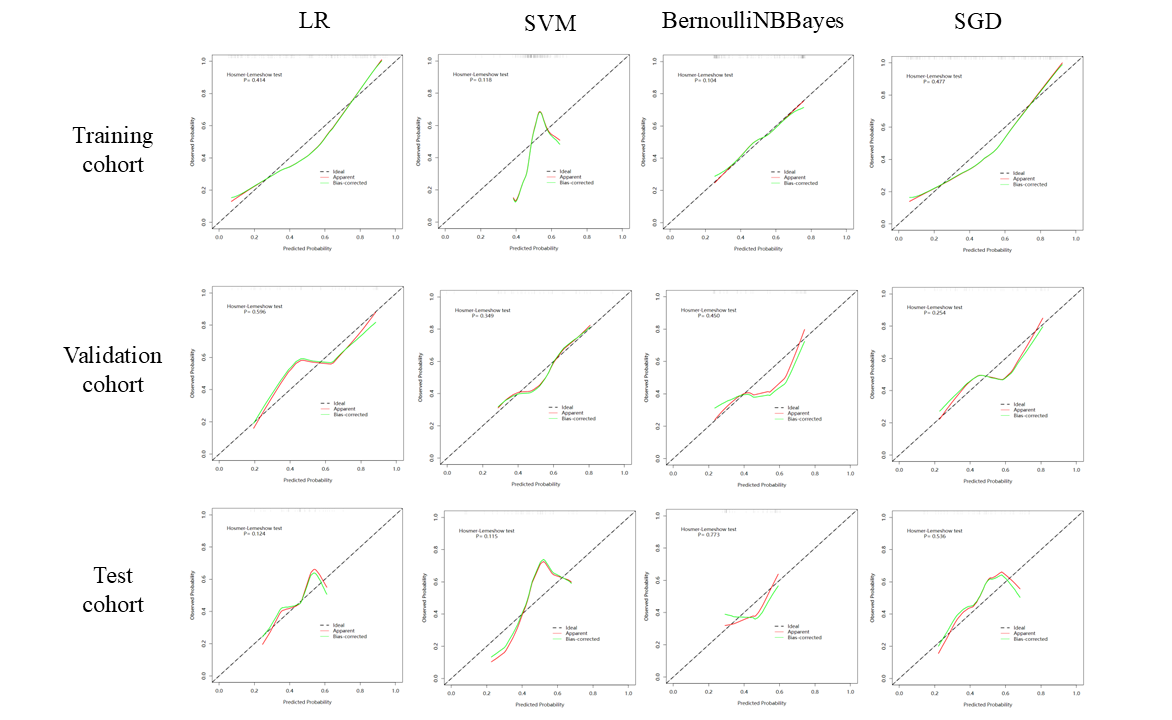

Supplement: Supplementary file 1 [file diagnostics-15-02722-s001.zip › Non-published Material/Supplementary Figure 2.tif]
